# Hantaviruses use the endogenous host factor P58^IPK^ to combat the PKR antiviral response

**DOI:** 10.1371/journal.ppat.1010007

**Published:** 2021-10-15

**Authors:** Zekun Wang, Songyang Ren, Qiming Li, Austin D. Royster, Lei lin, Sichen Liu, Safder S. Ganaie, Jianming Qiu, Sheema Mir, Mohammad A. Mir

**Affiliations:** 1 Joint National Laboratory for Antibody Drug Engineering, School of Basic Medical Sciences, Henan University, Kaifeng, China; 2 Western University of Health Sciences, Pomona, California, United States of America; 3 Department of Microbiology, Molecular Genetics and Immunology, University of Kansas Medical Center, Kansas City, Kansas, United States of America; The Scripps Research Institute, UNITED STATES

## Abstract

Hantavirus nucleocapsid protein (NP) inhibits protein kinase R (PKR) dimerization by an unknown mechanism to counteract its antiviral responses during virus infection. Here we demonstrate that NP exploits an endogenous PKR inhibitor P58^IPK^ to inhibit PKR. The activity of P58^IPK^ is normally restricted in cells by the formation of an inactive complex with its negative regulator Hsp40. On the other hand, PKR remains associated with the 40S ribosomal subunit, a unique strategic location that facilitates its free access to the downstream target eIF2α. Although both NP and Hsp40 bind to P58^IPK^, the binding affinity of NP is much stronger compared to Hsp40. P58^IPK^ harbors an NP binding site, spanning to N-terminal TPR subdomains I and II. The Hsp40 binding site on P58^IPK^ was mapped to the TPR subdomain II. The high affinity binding of NP to P58^IPK^ and the overlap between NP and Hsp40 binding sites releases the P58^IPK^ from its negative regulator by competitive inhibition. The NP-P58^IPK^ complex is selectively recruited to the 40S ribosomal subunit by direct interaction between NP and the ribosomal protein S19 (RPS19), a structural component of the 40S ribosomal subunit. NP has distinct binding sites for P58^IPK^ and RPS19, enabling it to serve as bridge between P58^IPK^ and the 40S ribosomal subunit. NP mutants deficient in binding to either P58^IPK^ or RPS19 fail to inhibit PKR, demonstrating that selective engagement of P58^IPK^ to the 40S ribosomal subunit is required for PKR inhibition. Cells deficient in P58^IPK^ mount a rapid PKR antiviral response and establish an antiviral state, observed by global translational shutdown and rapid decline in viral load. These studies reveal a novel viral strategy in which NP releases P58^IPK^ from its negative regulator and selectively engages it on the 40S ribosomal subunit to promptly combat the PKR antiviral responses.

## Author summary

Activation of PKR during virus infection shuts down the host translation machinery and creates an antiviral state to create obstacles for viral protein synthesis. Our results demonstrate that hantaviruses hijack an endogenous PKR inhibitor P58^IPK^ to combat the PKR antiviral response. The PKR remains associated with the host ribosomes to gain free access for eIF2α that enables the prompt shutdown of host translation apparatus during virus infection. The studies reported here reveal a novel mechanism by which hantavirus NP prevents PKR induced host translation shutoff in virus infected cells. NP dissociates the inactive Hsp40-P58^IPK^ complex and recruits the released P58^IPK^ to the 40S ribosomal subunit, a strategic PKR location. This tactic recruitment of P58^IPK^ rapidly inhibits PKR and prevents host interference in viral protein synthesis.

## Introduction

Hantaviruses are zoonotic negative sense RNA viruses in the *Hantaviridae* family, *Bunyavirales* order. Their genome is composed of three RNA segments S, M and L, which encode nucleocapsid protein (NP), glycoprotein precursor (GPC) and RNA dependent RNA polymerase (RdRp), respectively [1]. The GPC is post-translationally cleaved into two glycoproteins Gn and Gc. Humans are infected by the inhalation of aerosolized excreta from virus-infected rodents [2]. Recently human to human transmission has been reported with the Andes hantavirus species in South America [3]. Hantavirus infections cause Hemorrhagic fever with renal syndrome (HFRS) and Hantavirus cardiopulmonary syndrome (HCPS), having mortality rates of 15% and 40%, respectively [4]. Although 150,000 to 200,000 cases of hantavirus infection are annually reported worldwide, there is no antiviral therapeutic or FDA approved vaccine for this virus infection.

The virus-host interaction determines the outcome of a viral disease. The host type I interferon (IFN) response stimulates the expression of interferon stimulated genes (ISGs) [5]. The antiviral effector functions of ISGs promote the establishment of antiviral state in the host cell to create hurdles for virus replication [6]. Although the cytoplasmic tail domain of hantavirus Gn inhibits IFN induction during early stages of virus infection [7–9], a vigorous IFN and ISG expression is observed during later stages of hantavirus infection [10]. The delayed but robust IFN response fails to combat virus replication in hantavirus infected hosts [7,10,11], suggesting that hantaviruses have evolved strategies to counteract the ISGs’ antiviral effects.

Protein kinase R (PKR), one of the classical ISGs from the eIF2α-kinase family has a C-terminal kinase domain (KD) and an N-terminal dsRNA binding domain (dsRBD), composed of two tandem dsRNA binding motifs. The intermolecular interaction between dsRBD and KD maintains this enzyme in the latent form. Upon binding to dsRNA or its activator PACT protein, PKR undergoes a conformational change and forms a homo-dimer [12,13]. The dimerized PKR undergoes auto-phosphorylation to attain activity [14], which then plays diverse roles in the host antiviral defense. For example, the active PKR phosphorylates eIF2α to induce the host translation shutoff, aimed to interrupt the viral protein synthesis [15]. PKR also activates NF-κB to positively regulate the induction of interferon and inflammatory cytokines [16]. The activation of apoptotic pathways to limit virus replication and spread is yet another aspect of antiviral defense regulated by PKR [17]. The location of PKR inside the host cell might play a role in the diverse functions of PKR. Interestingly, PKR remains associated with the host cell ribosomes, especially the 40S ribosomal subunit [18,19]. The association with the ribosome has been proposed to facilitate the free access of PKR to its primary downstream target eIF2α, although the role of this strategic location in other antiviral functions of PKR remains unclear. Being a key antiviral host factor, viruses have evolved multiple strategies to antagonize PKR antiviral activity [20,21]. On the other hand, PKR activation is also strictly regulated by the host cell factors, such as, HIV-1 TAR RNA binding protein (TRBP), Glycoprotein p67, Nucleophosmin, Melanoma differentiation-associated gene-7 protein, Heat shock proteins Hsp90, Hsp70 and P58^IPK^ [14].

The P58^IPK^, an endogenous PKR inhibitor [22] from the tetratricopeptide repeat (TPR) family, contains nine tandemly arranged repeats (TPR1-9) of 34 amino acids at the N-terminus. The homology between its C-terminal domain and the J domain of the DnaJ heat shock proteins also makes P58^IPK^ a member of the HSP family of proteins [23]. The P58^IPK^ inhibits PKR by direct binding and interruption in PKR dimerization [22]. The PKR binding domain of P58^IPK^ has been mapped to its TPR6 motif [22]. Interestingly, the activity of P58^IPK^ is itself negatively regulated by its own inhibitor Hsp40, which forms an inactive complex with P58^IPK^ (22). We have previously reported that hantavirus NP interrupts PKR dimerization by an unknown mechanism to ensure continuous synthesis of viral proteins in the host cell [24]. Here we demonstrate a novel mechanism by which hantavirus NP releases P58^IPK^ from the clutches of Hsp40 and recruits it to the 40S ribosomal subunit to inhibit PKR dimerization. Our results demonstrate that NP binds to P58^IPK^ with higher affinity as compared to Hsp40. Both NP and Hsp40 share a common binding site on P58^IPK^. Our results suggest that NP competes with Hsp40 for binding to P58^IPK^, leading to the dissociation of Hsp40-P58^IPK^ complex. The resulting NP-P58^IPK^ complex is selectively recruited to the 40S ribosomal subunit by the direct interaction between NP and the ribosomal protein S19 (RPS19), a structural component of the 40S ribosomal subunit. NP has distinct binding sites for RPS19 and P58^IPK^. The simultaneous binding of NP to both the P58^IPK^ and RPS19 facilitates the prompt recruitment of P58^IPK^ to the 40S ribosomal subunit. Interruption in the recruitment process prevents PKR inhibition in cells. Collectively, our studies reveal a novel mechanism by which hantavirus NP releases P58^IPK^ from the clutches of its negative regulator and recruits it on the 40S ribosomal subunit to efficiently counteract the PKR antiviral responses.

## Results

### NP binds to P58^IPK^ and inhibits the PKR

**Fig 1:**
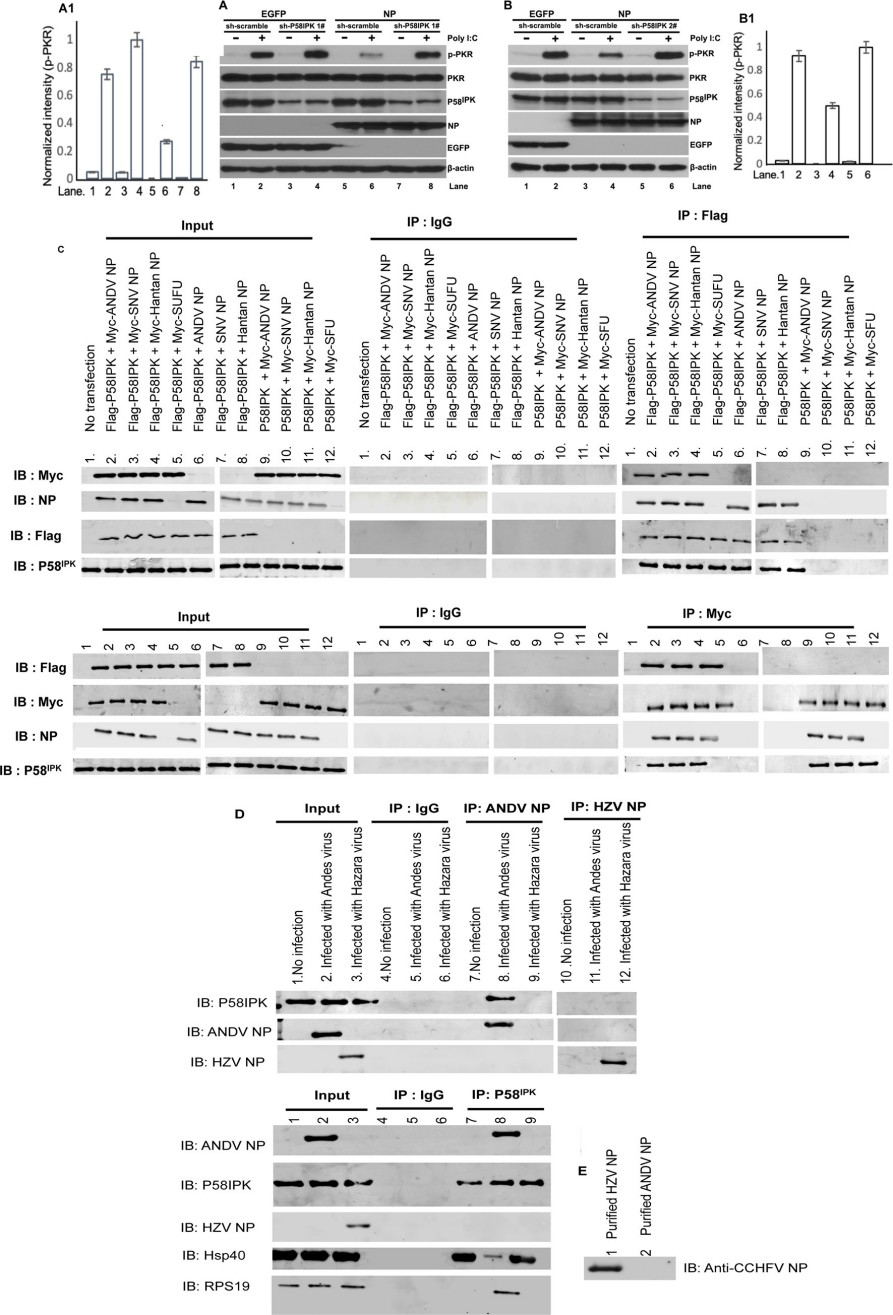
NP recruits P58^IPK^ to inhibit PKR activation. **A and B.** HEK293T stable cell lines expressing either EGFP or SNV NP were transduced with lentivirus for the stable expression of either scrambled shRNA or shRNA#1 (panel A) or shRNA#2 (panel B) against P58^IPK^. The shRNAs #1 and #2 were targeted to different regions of the P58^IPK^ mRNA sequence. Cells were either mock transfected or transfected with Poly I:C (400 ng/ml) for three hours before harvesting. Cell lysates were examined for the expression of phosphorylated PKR (p-PKR), total PKR, P58^IPK^, NP, EGFP and β-actin by western blot analysis using appropriate antibodies. See Materials and Methods for antibodies used **A1 and B1.** The band intensities of p-PKR were quantified by imageJ and normalized related to the intensity in lane 2. The normalized intensity values were plotted verses lane number. The panels A1 and B1 represent the relative p-PKR band intensities from panels A and B, respectively. The standard deviations shown by error bars were calculated from two or more independent experiments. The significance of the difference was calculated by *t* test, as previously reported [24]. **C.** HEK293T cells were co-transfected with plasmids expressing the proteins shown in lanes 1–12. Cell lysates were immunoprecipitated (IP) with the antibodies shown in the figure, followed by western blot analysis (IB) of the immunoprecipitated material using the antibody shown in the figure. **D**. HUVEC cells were infected with either Andes virus or hazara virus. Cell lysates from infected cells were immunoprecipitated (IP) by the antibodies shown in the figure, except the IP of HZV NP was carried out using anti-CCHFV NP antibody as discussed in the text. The immunoprecipitated lysates were examined by western blot analysis (IB) using the antibodies shown in the figure. **E**. The bacterially expressed and purified HZV NP and ANDV NP were examined by western blot analysis using rabbit polyclonal anti-CCHFV antibody from abcam (ab190657).

We previously reported that hantavirus NP inhibits PKR activation by inhibiting its dimerization and subsequent autophosphorylation. The inactive PKR fails to phosphorylate its downstream targets, such as, eIF2α. To determine whether NP uses P58^IPK^ to inhibit the activation of PKR, we knocked down P58^IPK^ by shRNA in HEK293T cells, stably expressing either NP from SNV (Sin Nombre virus) or EGFP as negative control. The cells were transfected with poly I:C three hours before harvesting to trigger PKR activation, and were examined by western blot analysis for the phosphorylation of PKR. It is evident from (Fig 1A and 1A1) that cells stably expressing NP failed to proficiently inhibit poly I:C induced PKR autophosphorylation due to the down regulation of P58^IPK^ (compare lanes 6 and 8). The results were confirmed by using another shRNA targeted to a different region of P58^IPK^ mRNA (Fig 1B and 1B1, compare lanes 4 and 6). This experiment suggests that NP requires P58^IPK^ to proficiently inhibit the PKR. Unlike many other hantavirus NPs the NP from SNV does not block the RIGI/MDA5 mediated interferon β pathway [25,26], suggesting that potential inhibition of PKR activation through this route is less likely. To determine whether NP interacts with P58^IPK^, HEK293T cells were co-transfected with plasmids expressing FLAG tagged P58^IPK^ and Myc tagged NP from either SNV or ANDV (Andes virus) or Hantaan virus or Myc tagged SUFU (suppressor of fused homolog) protein of chicken origin as negative control (Fig 1C lanes 2–5). The molecular weight of SUFU protein is similar to hantavirus N protein. Similarly, cells were co-transfected with plasmids expressing FLAG tagged P58^IPK^ and NP from either SNV or ANDV or Hantaan virus devoid of any tag (Fig 1C, lanes 6–8). Cells were also co-transfected with plasmids expressing P58^IPK^ devoid of any tag and Myc tagged NP from either SNV or ANDV or Hantaan virus or Myc tagged SUFU protein (Fig 1C, lanes 9–12). Cell lysates were immunoprecipitated using either anti-FLAG antibody or IgG as control. An examination of the immunoprecipitated material by western blot analysis using anti-Myc antibody revealed an interaction between P58^IPK^ and NP from SNV, ANDV and Hantaan virus (Fig 1C). The reverse immunoprecipitation of cell lysates using anti-Myc antibody and examination of immunoprecipitated material by western blot analysis using anti-FLAG antibody further confirmed the results. The interaction of P58^IPK^ with the NPs of all three hantaviruses (SNV, ANDV and Hantaan virus) is consistent with their previously reported PKR inhibition [24].

To further confirm the P58^IPK^-NP interaction, HUVECs were infected with either ANDV or Hazara virus (HZV), a tick borne orthonairovirus having tri-segmented negative sense RNA genome similar to hantaviruses. HZV is used as model virus for the study of Crimean congo hemorrhagic fever virus (CCHFV), a BSL 4 orthonairovirus that causes significant human disease. HUVECs were used because these cells are permissive to both hantavirus and HZV infection. The cell lysates were immunoprecipitated using either anti-ANDV NP antibody or IgG or anti-CCHFV NP antibody that cross reacts with HZV NP due to significant sequence homology between CCHFV and HZV NPs. We confirmed the cross reactivity of anti-CCHFV NP antibody with bacterially expressed and purified HZV NP (Fig 1E). These anti-CCHFV NP antibodies have been previously used for HZV NP studies [27]. The cross-reactive CCHFV antibodies were used because the antibodies for HZV NP are not commercially available. Western blot analysis of the immunoprecipitated material using anti-P58^IPK^ antibody confirms the interaction between P58^IPK^ and ANDV NP in infected cells (Fig 1D). The reverse immunoprecipitation of cell lysates using anti-P58^IPK^ antibody, followed by Western blot analysis of the immunoprecipitated material using either anti-ANDV NP or anti-CCHFV NP antibodies further confirms the P58^IPK^-ANDV NP interaction during the course of infection (Fig 1D).

### NP releases P58^IPK^ from its negative regulator Hsp40

**Fig 2:**
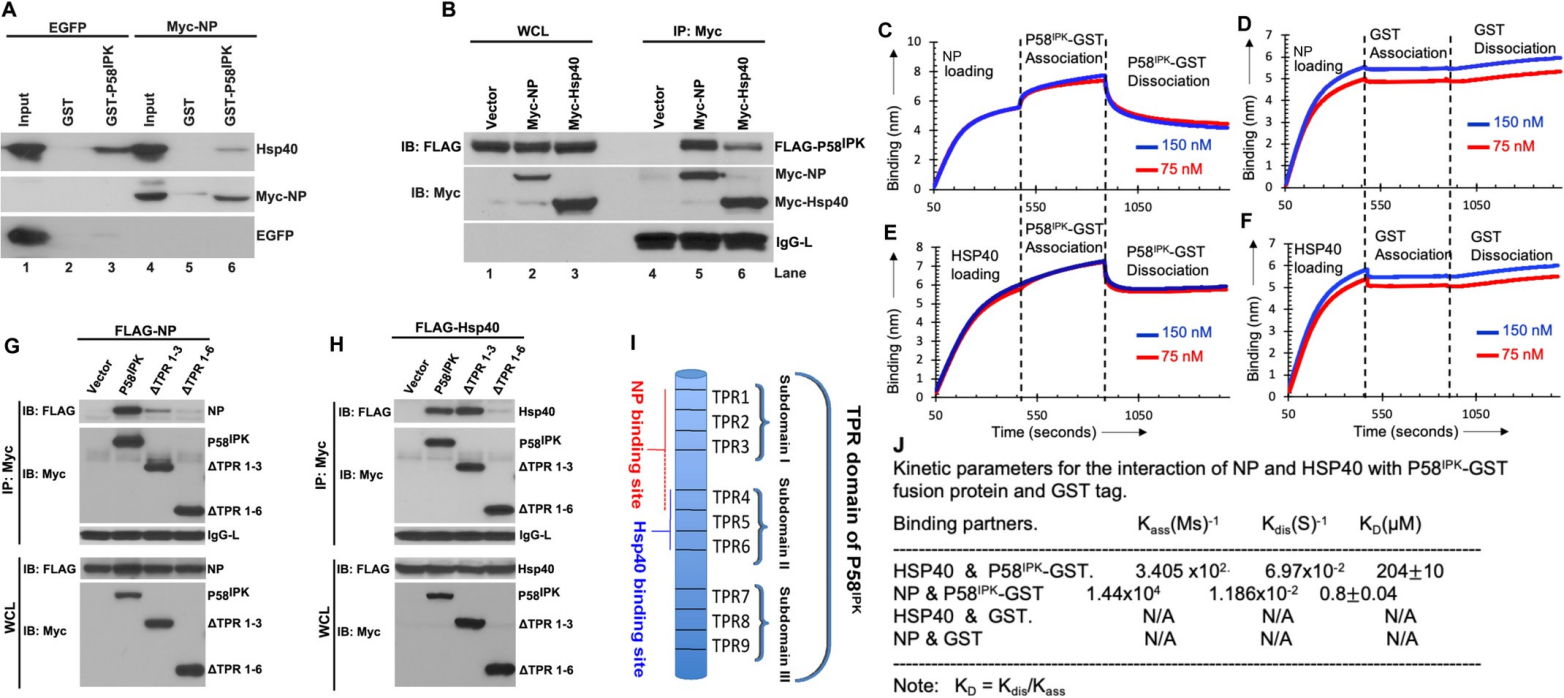
NP releases P58^IPK^ from its negative regulator Hsp40. **A**. Bacterially expressed and purified GST or GST-P58^IPK^ fusion proteins were incubated with Glutathione Sepharose beads. The beads were washed and further incubated with the lysates from HEK293T cells, stably expressing either EGFP (lanes 1–3) or Myc-NP (lanes 4–6). The bound material eluted from washed beads was examined by western blot analysis using appropriate antibodies. Input represents the whole cell lysate. **B.** HEK293T cells were co-transfected with plasmids expressing FLAG-P58^IPK^ along with either Myc-Hsp40 or Myc-NP. The cell lysates were immunoprecipitated using anti-Myc antibody. The western blot analysis of the whole cell lysate (WCL) (lanes 1–3) and immunoprecipiated material (lanes 4–6) was carried out using appropriate antibodies. **C, D, E and F**. Representative BLI sensograms showing over time association and dissociation of N protein with GST-P58^IPK^ (C), N protein with GST tag (D), HSP40 with GST-P58^IPK^ (E) and HSP40 with GST tag (F). The sensograms were generated at two concentrations of GST-P58^IPK^ and GST tag, shown by red and blue color in panels C-F (see materials and methods for details). **G.** HEK293T cells were cotransfected with plasmids expressing FLAG-NP or Myc tagged wild type P58^IPK^ or P58^IPK^ mutants lacking either subdomain I composed of TPR repeats 1–3 (ΔTPR1-3) or both subdomains I and II composed of TPR repeats 1–6 (ΔTPR1-6). Cell lysates were Immunoprecipitated using anti-Myc antibody. Western blot analysis of the whole cell lysate (WCL) or immunoprecipitated material was carried out using either anti-FLAG or anti-Myc antibodies, as shown. **H**. The experiment shown in this panel was carried out exactly as in panel C except FLAG-NP was replaced with FLAG-Hsp40. **I**. Schematic representation of TPR domain of P58^IPK^. **J**. The kinetic profiles from panels C-F were analyzed for the calculation of binding parameters, as mentioned in Materials and Methods. Five percent of the calculated K_D_ value was added as standard deviation.

In the absence of viral infection or cell stress, the P58^IPK^ forms an inactive complex with its negative regulator heat shock protein Hsp40. Since NP binds to P58^IPK^ (Fig 1), we asked whether NP affects the binding of Hsp40 to P58^IPK^, which might lead to the dissociation of inactive P58^IPK^-Hsp40 complex. HUVEC cell lysates from ANDV or HZV infected cells were immunoprecipitated with anti-P58^IPK^ antibody, followed by western blot analysis using anti-Hsp40 antibody. It is evident from Fig 1D that Hsp40-P58^IPK^ interaction was significantly impacted in ANDV infected cells in comparison to HZV or un-infected control cells (compare lanes 7,8 and 9 in Fig 1D). To further confirm that NP impacted the Hsp40-P58^IPK^ interaction, bacterially expressed and purified GST-P58^IPK^ fusion protein was immobilized on Glutathione Sepharose beads. The beads were incubated with HEK293T cell lysates stably expressing either EGFP or NP, as mentioned in Materials and Methods. An examination of the GST pull-down material by western blot analysis revealed that Hsp40 strongly bound to GST-P58^IPK^ in control cell lysates expressing EGFP. However, the interaction was impacted in cell lysates expressing NP (compare lane 6 with lane 3 in Fig 2A). The co-purification of both NP and Hsp40 with the GST-P58^IPK^ suggests that NP likely competed with the Hsp40 for binding to GST-P58^IPK^. We next compared the binding of both NP and Hsp40 with the P58^IPK^ in cells. HEK293T cells were co-transfected with plasmids expressing FLAG tagged P58^IPK^ along with either Myc tagged NP or Myc tagged Hsp40. Cell lysates were immunoprecipitated with anti-Myc antibody and the binding of FLAG tagged P58^IPK^ was examined by western blot analysis using anti-FLAG antibody. The strong co-purification of Myc-NP with FLAG-P58^IPK^ suggests that NP likely binds to P58^IPK^ more tightly as compared to Hsp40 (compare lanes 5 and 6 in Fig 2B). It is possible that both NP and Hsp40 share a common binding site on P58^IPK^ and NP competes with Hsp40 for binding to P58^IPK^. Further studies will be required to demonstrate if NP competitively inhibits the binding of Hsp40 to P58^IPK^. It is equally possible that binding of NP induces a conformational change in P58^IPK^ and conformationally altered P58^IPK^ does not bind to Hsp40.

We next quantified the binding affinity of P58^IPK^ with both NP and Hsp40 using biolayer interferometry on BLITZ (ForteBio) instrument, as previously reported [28,29]. Briefly, the bacterially expressed and purified C-terminally His tagged NP and Hsp40 were immobilized on Ni-NTA biosensor, following by the examination of association and dissociation kinetics of purified GST- P58^IPK^ and GST (control) with the immobilized protein (see materials and methods for details). The representative kinetic profiles are shown in Fig 2C–2F and the binding data is shown in Fig 2J. It is evident from Fig 2C–2F that unlike GST control the GST-P58^IPK^ showed interaction with both NP and Hsp40. The GST-P58^IPK^ bound to NP with slower on-rate compared to Hsp40. However, the dissociation kinetics of GST- P58^IPK^ -NP complex was slower compared to GST- P58^IPK^-Hasp40 complex. Analysis of the kinetic data reveled that GST- P58^IPK^ bound to NP and Hsp40 with the dissociation constants (K_D_) of ~ 0.8 μM and ~ 204 μM, respectively (Fig 2J). Similar binding affinities have been reported between most regulatory and signaling proteins [30–33]. This experiment clearly demonstrates that P58^IPK^ binds to NP with 250-fold stronger affinity compared to Hsp40.

We mapped the binding sites for NP and Hsp40 on P58^IPK^ using deletion mutation and immunoprecipitation analysis. The N-terminal TPR domain of P58^IPK^ is composed of nine TPR motifs (TPR1-TPR9). The structural motifs TPR1-TPR3, TPR4-TPR6, TPT7-TPR9 comprise three subdomains I, II and III, respectively (Fig 2I). Two P58^IPK^ deletion mutants lacking either subdomain I (TPR1-TPR3) or both subdomains I and II (TPR1-TPR6) were expressed as N-terminally Myc tagged fusion proteins in HEK293T cells along with FLAG tagged NP. Cell lysates were immunoprecipitated with anti-Myc antibody and the binding of FLAG-NP was determined by western blot analysis using anti-FLAG antibody. It is evident from Fig 2G that deletion of subdomain I (ΔTPR1-3) significantly inhibited the binding of P58^IPK^ to NP, which was further compromised by the deletion of both the subdomain I and II (ΔTPR1-6). This observation suggests that NP binding site is located in the subdomains I and II, although the subdomain I might play a major role in the binding. A similar experiment was performed to map the binding site of Hsp40 on P58^IPK^. It is evident from Fig 2H that deletion of subdomain I did not affect the binding of P58^IPK^ to Hsp40. However, the binding was significantly compromised by the deletion of both the subdomains I and II, suggesting that Hsp40 binding site is most likely located in the subdomain II. Taken together, the results from Fig 2 demonstrate that NP binding site likely overlaps with the Hsp40 binding site on P58^IPK^. Since NP binds to P58^IPK^ with higher affinity as compared to Hsp40 (Fig 2C–2F), it is possible that NP might competitively block the binding of Hsp40 to P58^IPK^, although further studies are required to prove a possible competitive inhibition. A weaker possibility that a host factor indirectly mediates the interaction between NP and P58^IPK^ *in vivo* cannot be ruled out.

### The binding of NP to P58^IPK^ is essential but not sufficient to inhibit PKR

**Fig 3:**
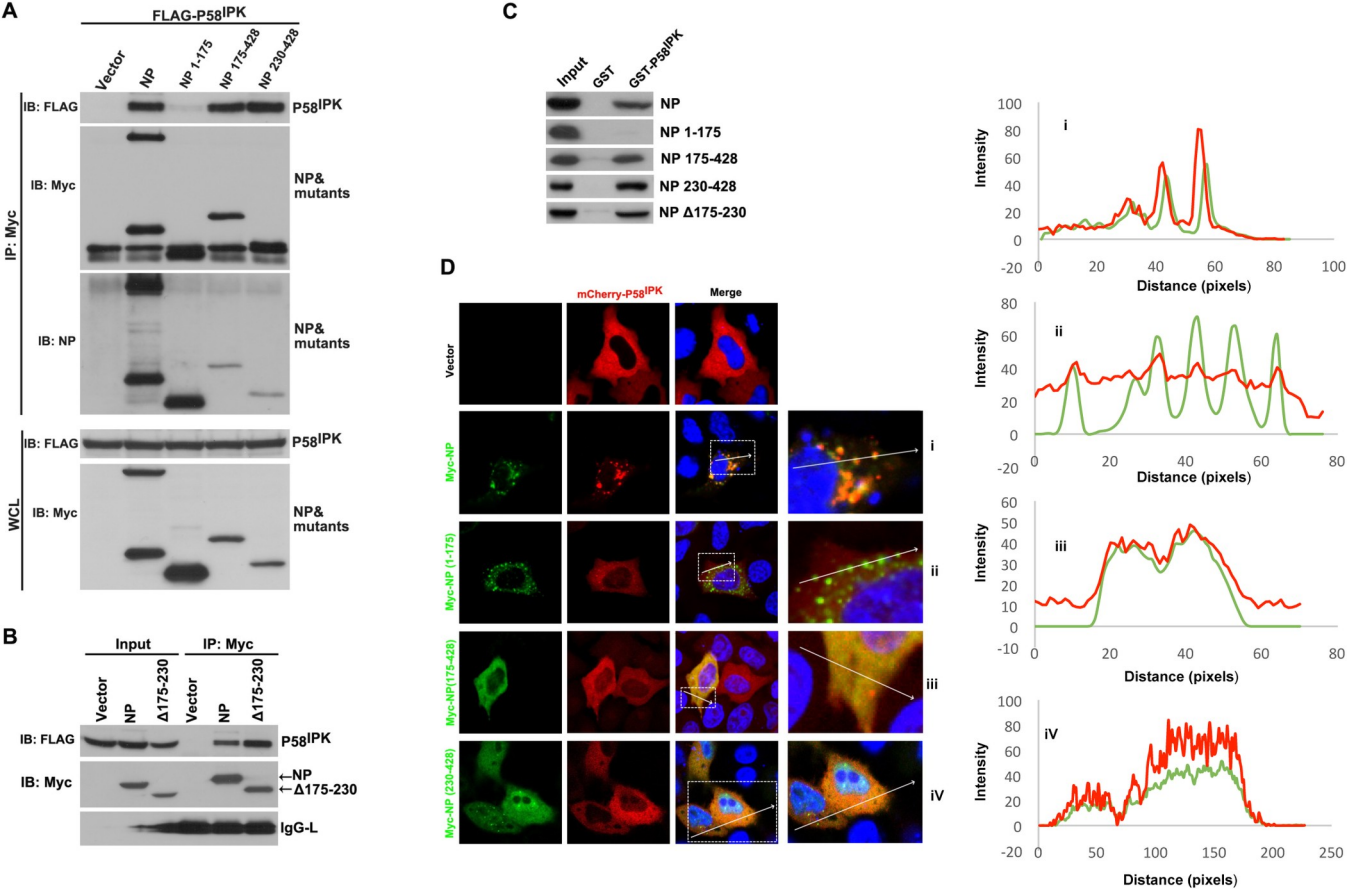
NP harbors a P58^IPK^ binding site at the C-terminus. **A.** HEK293T cells were co-transfected with plasmids expressing FLAG-P58^IPK^ along with either wild type Myc-NP or NP truncation mutants NP1-175 or NP175-428 or NP230-428 fused to the Myc tag. Western blot analysis of the immunoprecipitated material or whole cell lysate (WCL) was carried out using appropriate antibodies. The western blot analysis of the immunoprecipitated material using anti-Myc antibody did not show NP1-175 and NP230-428 mutants due their size coinciding with light chain antibody (2^nd^ from top). The blot was re-probed using anti-NP antibody, which showed the mutants NP1-175 and NP230-428 (3^rd^ from top). The lower band in NP lane shows a degradation product. **B.** The experiment shown in panel A was repeated here to test another NP mutant lacking the RNA binding domain (NPΔ175–230) and results were compared with wild type NP. **C**. Bacterially expressed and purified GST or GST-P58^IPK^ was immobilized on Glutathione Sepharose beads. Beads were washed and incubated with HEK293T cell lysates containing wild type NP or NP mutants. The material eluted from washed beads was examined by western blot analysis using anti-Myc antibody. **D**. HeLa cells were cotransfected with plasmids expressing mCherry-P58^IPK^ along with either wild type NP or NP truncation mutants fused with Myc tag. The Myc tagged proteins were probed with anti-Myc antibody and visualized using a secondary antibody conjugated with Alexa Fluor 488. The boxed area in the merge panels were magnified and presented sidewise with labels I, ii, iii and iv. Using the ImageJ software, a line was drawn through the two or more puncta and the fluorescence intensity under the line is represented in the sidewise graphs corresponding to panels I, ii, iii and iv, as shown. The red line is the fluorescence intensity of mCherry-P58IPK, and the green line represents the intensity of the Alexa Fluor 488. Unlike panel ii, the red and green fluorescence signals in panels I, iii and iv show strong colocalization as evidenced by the fact that both signals are significantly above the background and increase at the same location.

To further gain insights into the molecular mechanisms, we mapped the P58^IPK^ binding domain on NP using immunoprecipitation and western blot analysis. HEK293T cells were co-transfected with plasmids expressing FLAG-P58^IPK^ along with either wild type Myc-NP or Myc-NP truncation mutants containing N-terminal 175 amino acids (NP1-175) or C-terminal 175–428 amino acids (NP175-428) or C-terminal 230–428 amino acids (NP230-428). The cell lysates were immunoprecipitated using anti-Myc antibody. Western blot analysis of immunoprecipitated material by anti-FLAG antibody revealed that N-terminal 175 amino acids didn’t bind to P58^IPK^ (Fig 3A). In comparison, both C-terminal mutants (NP175-428 and NP230-428) showed strong binding to P58^IPK^, demonstrating that P58^IPK^ binding site is located at the C-terminus of NP. A similar analysis revealed that NP mutant lacking the RNA binding domain (amino acids 175–230) bound to P58^IPK^ similar to wild type NP (Fig 3B). The results from Fig 3A and 3B were further verified by GST pull-down assay. Bacterially expressed and purified GST-P58^IPK^ was immobilized on Glutathione Sepharose beads. The beads were incubated with HEK293T cell lysates expressing either wild type Myc-NP or Myc-NP truncation mutants. An examination of the eluted material from washed beads by western blot analysis using anti-Myc antibody further confirmed that P58^IPK^ binding site is located at the C-terminus of NP (Fig 3C).

**Fig 4:**
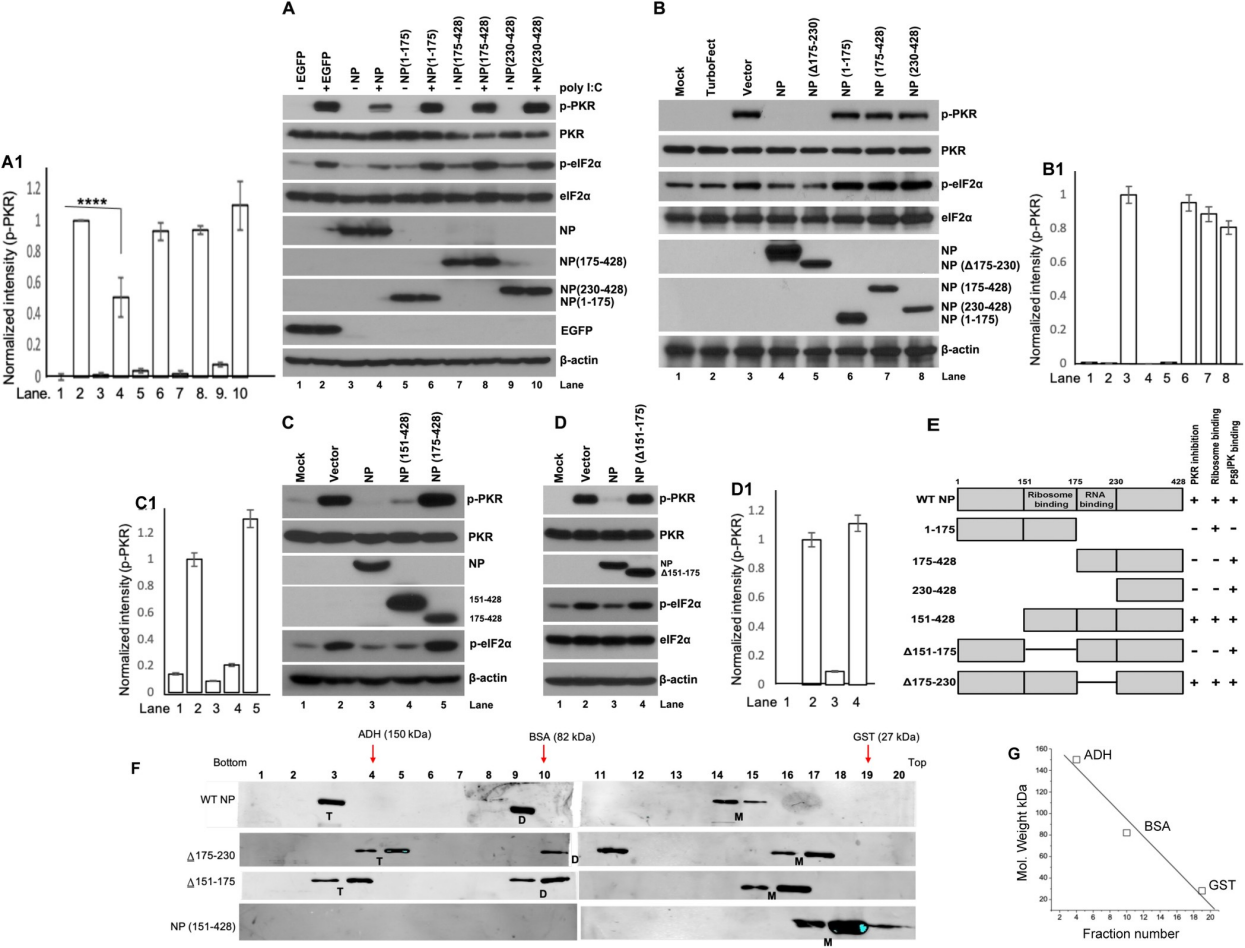
NP requires both RPS19 and P58^IPK^ binding domains to inhibit PKR. **A**. HEK293T stable cell lines expressing either EGFP or wild type NP or NP truncation mutants were either mock transfected or transfected with Poly I:C three hours before harvesting to stimulate PKR autophosphorylation. Cells were harvested and the expression of indicated proteins was examined by western blot analysis using appropriate antibodies. **A1**. The band intensities of p-PKR in panel A were quantified by imageJ and normalized intensity values were plotted verses lane number, as discussed in panel Fig 1. **B**. HEK293T cells were either mock transfected or transfected with the transfection reagent (TurboFect) alone, or TurboFect containing empty vector (pcDNA3.1) or expression vectors expressing either wild type NP or NP deletion mutant lacking the RNA binding domain (NPΔ175–230) of NP truncation mutants (NP1-175, NP175-428 or NP230-428). Cells were harvested and examined for the expression of indicated proteins using appropriate antibodies. **B1**. The band intensities of p-PKR in panel B were quantified by imageJ and normalized intensity values were plotted verses lane number. Error bars are the ten percent of the normalized intensity value. **C and D.** HEK293T cells were either mock transfected or transfected with empty vector or expression vectors expressing wild type NP or NP truncation mutants NP151-428, NP175-428 (panel C) or deletion mutant lacking the RPS19 binding domain (NPΔ151–175) (Panel D). Cell lysates were examined for the expression of indicated proteins using appropriate antibodies. **C1 and D1**. The band intensities of p-PKR in panel C & D were quantified by imageJ and normalized intensity values were plotted verses lane number. Error bars are the ten percent of the normalized intensity value. **E**. Pictorial representation of wild type NP and NP mutants. The horizontal line represents the deleted region from the amino acid sequence. **F**. Sucrose density gradient centrifugation to examine the oligomerization forms of wild type NP and its mutants. The proteins (GST, BSA, ADH, W.t NP, Δ175–230, Δ151–175, NP(151–428)) were fractionated on 5–50% sucrose gradient and gradient fractions were examined by western blot analysis. Note: M, D and T represents the monomeric, dimeric and trimeric forms of the protein, based on the Molecular weight determination using Fig 4G. **G**. A plot of molecular weight of GST, BSA and ADH verses the fraction number in which these proteins eluted in the gradient. The fractions in which these proteins eluted in the gradient are also shown by red arrows in Fig 4F.

We next used confocal microscopy to visualize the location of P58^IPK^ in cells expressing either wild type NP or NP truncation mutants. HeLa cells were cotransfected with plasmids expressing mCherry-P58^IPK^ fusion protein along with wild type Myc-NP or Myc-tagged NP mutants (Fig 3D). Wild type NP or NP mutants fused to Myc tag were visualized using anti-Myc primary antibody and secondary antibody conjugated with Alexa Flour 488. Unlike Myc-NP1-175 mutant, both wild type and NP mutants (NP175-428 and NP230-428) showed strong colocalization with mCherry-P58^IPK^, consistent with the results from Fig 3A and 3B. We have previously reported that NP binds to the 40S ribosomal subunit via the ribosomal protein S19, a structural component of the 40S ribosomal subunit. NP harbors the RPS19 binding domain, located in the region from 151–175 amino acids. It is evident from Fig 3D that wild type NP and NP1-175 mutant having the RSP19 binding domain show perinuclear punctuate morphology, probably due to the association with endoplasmic reticulum associated ribosomes. The NP mutants NP230-428 and NP175-428 lacking the RSP19 binding domain were located all over the cytoplasm, and the punctuate morphology was completely lost. The mCherryP58^IPK^ localized to cytoplasm in cells lacking NP expression (Fig 3D). NP mutants are shown in schematic presentation in Fig 4E. However, mCherryP58^IPK^ adopted a striking punctuate perinucler morphology in NP expressing cells, resembling to the morphology of wild type NP. Although mCherry-P58^IPK^ strongly colocalized with NP mutants (NP175-428 and 230–428), its parental cytoplasmic morphology was retained. These observations suggest that wild type NP likely targets mCherry-P58^IPK^ to the 40S ribosomal subunit.

To determine whether the binding of NP to P58^IPK^ was sufficient to inhibit PKR, HEK293T stable cell lines expressing either wild type NP or NP truncation mutants were transfected with poly I:C three hours before harvesting to trigger PKR activation. Cell lysates were examined by western blot analysis to monitor PKR autophosphorylation (Fig 4A and 4A1). As expected, the NP1-175 mutant, deficient in binding to P58^IPK^, failed to inhibit PKR autophosphorylation. Surprisingly, NP175-428 and NP230-428 mutants that strongly bind to P58^IPK^ also failed to inhibit PKR autophosphorylation, suggesting that binding to P58^IPK^ may be required but is not sufficient to inhibit PKR. It has been reported that transient plasmid transfection triggers PKR activation in cells [34,35]. To further verify the results from Fig 4A, HEK293T cells were transiently transfected with either empty vector or plasmids expressing either wild type NP or NP truncation mutants used in Fig 4A or NP deletion mutant lacking the RNA binding domain (NPΔ175–230). An examination of cell lysates by western blot analysis revealed that transfection of empty vector induced PKR activation, which was inhibited by the expression of wild type NP and NP mutant lacking RNA binding domain (Fig 4B and 4B1), consistent with our previously reported similar observations [24]. In addition, the C-terminal NP mutants (NP175-428 and NP230-428) that bind to P58^IPK^ again failed to inhibit PKR, thus verifying the results from Fig 4A. The strategy from Fig 4B was used to test the activity of an additional NP mutant (NP151-428), which contains intact RPS19 binding domain [36]. Surprisingly, this mutant inhibited PKR activation comparable to wild type NP (Fig 4C and 4C1). Deletion of RPS19 binding domain abolished the activity of NP to inhibit PKR (Fig 4D). Taken together, the results from Fig 4 demonstrate that NP requires both P58^IPK^ and RPS19 binding domains to inhibit PKR.

### Oligomerization of NP is not required to inhibit PKR activation

Hantavirus NP forms dimers and trimers upon expression, and the resulting monomeric, dimeric and trimeric forms of NP remain in dynamic equilibrium. Recently, the X-ray crystallographic studies have demonstrated that C-terminal linker and helix regions connecting the N-terminal coiled-coil domain and core region are essential for NP oligomerization [37]. Moreover, the electron microscopic (EM) visualization of native ribonucleoprotein complexes (RNPs) extracted from the virions revealed that a monomer-sized NP-RNA complex is the building block of the viral RNP [37], demonstrating that monomeric forms of NP are functional. We examined the oligomerization status of wildtype NP and its mutants on sucrose density gradient to determine whether oligomerization of NP is required to inhibit PKR activation. As shown in Fig 4F, the wildtype NP (~53 KDa) formed dimers (~106 KDa) and trimers (~159 KDa), and inhibited the PKR activation (Fig 4A, 4B and 4C). However, the NP mutant (NPΔ151–175) formed dimers and trimers similar to wildtype NP (Fig 4F) but failed to inhibit PKR activation (Fig 4D and 4D1). In comparison, the NP mutant (NP151-428) didn’t form the dimers and trimers (Fig 4F) due to the lack of N-terminal Coiled-coil domain, required for oligomerization, but inhibited the PKR activation (Fig 4C and 4C1). This clearly demonstrates that oligomerization of NP is not required to inhibit PKR activation.

### NP recruits P58^IPK^ to the 40S ribosomal subunit with the assistance of ribosomal protein RPS19

**Fig 5:**
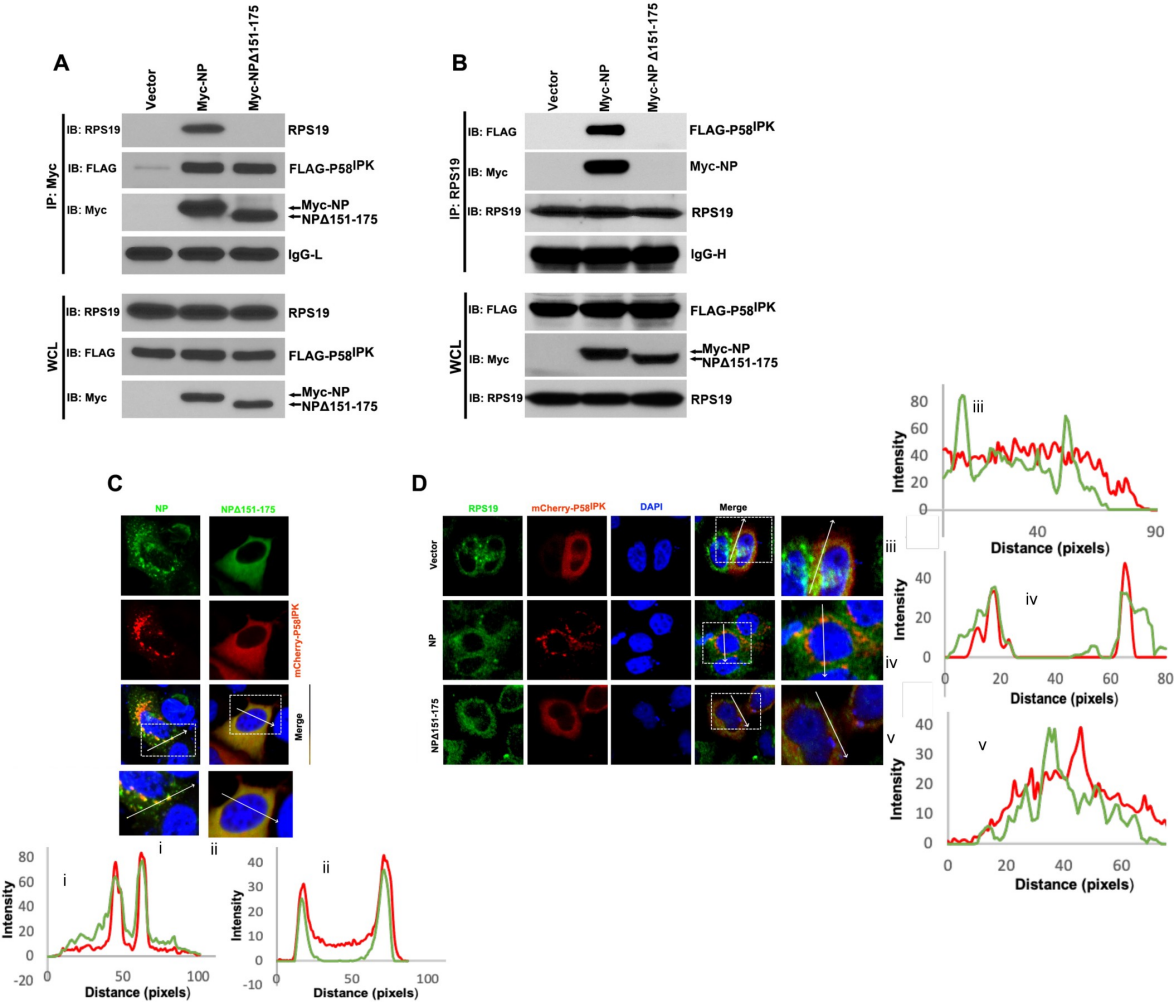
NP recruits P58^IPK^ to the 40S ribosomal subunit. **A.** HEK293T cells were cotransfected with plasmids expressing FLAG-P58^IPK^ along with either Myc-NP or Myc-NPΔ151–175 deletion mutant. Cell lysates were immunoprecipitated with anti-Myc antibody. The whole cell lysate (WCL) or immunoprecipitated material was examined by western blot analysis (IB) using either anti-RPS19 or anti-FLAG tag or anti-Myc tag antibodies. **B.** HEK293T cells were transfected as mentioned in panel A. The cell lysates were immunoprecipitated with RPS19 antibody. The whole cell lysate (WCL) or immunoprecipitated material was examined by western blot analysis (IB) using either anti-RPS19 or anti-FLAG tag or anti-Myc tag antibodies. **C.** HeLa cells were cotransfected with plasmids expressing mCherry-P58^IPK^ along with either Myc-NP or Myc-NPΔ151–175 deletion mutant. The cells were examined by confocal microscope. The wild type NP and NPΔ151–175 mutant were visualized using anti-Myc primary antibody and a secondary antibody conjugated with Alexa Fluor 488. Quantification of the boxed zoomed area in the merged panels was carried out using imageJ as described in Fig 3D and presented as line graph at the bottom. The red and green fluorescence signals show strong colocalization evidenced by the significant increase in both fluorescence signals at the same location above the background. **D.** HeLa cells were cotransfected with mCherry-P58^IPK^ expressing plasmid along with either empty vector or plasmids expressing wild type NP or NPΔ151–175 deletion mutant. The cells were examined by confocal microscope. The RPS19 was visualized using a primary anti-RPS19 antibody and a secondary antibody conjugated with Alexa Fluor 488. Quantification of the boxed zoomed area in the merged panels was carried out using imageJ as described in Fig 3D and presented sidewise as line graph. Unlike panels iii and v, the red and green fluorescence signals in panel iv show colocalization evidenced by the increase in both fluorescence signals at the same location above the background.

The observations from Figs 4 and 3D suggested that NP likely recruits P58^IPK^ to the 40S ribosomal subunit, a previously known location of PKR. To test this hypothesis, HEK293T cells were co-transfected with plasmids expressing FLAG-P58^IPK^ along with either Myc-NP or Myc-NPΔ151–175 deletion mutant. Cell lysates were immunoprecipitated with anti-Myc antibody. An examination of the immunoprecipitated material by western blot analysis revealed that wild type NP co-purified with both RPS19 and FLAG-P58^IPK^, demonstrating the possible recruitment of P58^IPK^ to the 40S ribosomal subunit (Fig 5A). In comparison, the NPΔ151–175 mutant showed interaction with P58^IPK^ but failed to bind RPS19, as expected. To further verify whether NP recruits P58^IPK^ to the 40S ribosomal subunit, HEK293T cells were transfected as mentioned above. The 40S ribosomal subunit RPS19 was pulled down using anti-RPS19 antibody. An examination of the pulled-down material by western blot analysis revealed that RPS19 interacted with P58^IPK^ in NP expressing cells (Fig 5B). This interaction was not observed in cells transfected with empty vector or a plasmid expressing NPΔ151–175 mutant (Fig 5B). These results suggest that NP recruits P58^IPK^ to the 40S ribosomal subunit. To confirm that NP recruits P58^IPK^ to the 40S ribosomal subunit in virus infected cells, HUVEC cell lysates from ANDV or HZV infected cells were immunoprecipitated with anti-P58^IPK^ antibody. An examination of the immunoprecipitated material revealed that P58^IPK^ in ANDV infected cells copurified with both NP and RPS19, demonstrating the recruitment of P58^IPK^ to the 40S ribosomal subunit by the NP (Fig 1D).

We used confocal microscopy to further evaluate this hypothesis. It is evident from Fig 5C, that wild type NP shows a perinuclear punctate morphology, which is lost due to the deletion of RPS19 binding domain, as NPΔ151–175 deletion mutant is located all over the cytoplasm. This observation suggests that punctuate perinuclear morphology of wild type NP is likely due to its association with the 40S ribosomal subunit via RPS19. Although P58^IPK^ strongly colocalized with both wildtype NP and NPΔ151–175 deletion mutant, the parental cytoplasmic morphology of P58^IPK^ was altered into punctuate perinuclear morphology in NP expressing cells, resembling the morphology of wild type NP (Fig 5C), consistent with similar results from Fig 3D. This observation again suggests that NP likely recruits P58^IPK^ to the 40S ribosomal subunit. To verify this observation, we examined the localization of mCherry-P58^IPK^ fusion protein with RPS19, a structural constituent of the 40S ribosomal subunit, in cells expressing either wild type NP or NP deletion mutant. It is evident from Fig 5D that mCherry-P58^IPK^ does not colocalize with RPS19 in cells lacking NP expression, suggesting that mCherry-P58^IPK^ by itself does not bind to ribosomes. The colocalization between mCherry-P58^IPK^ and RPS19 in NP expressing cells suggests that NP specifically mediates the interactions between P58^IPK^ and the 40S ribosomal subunit. This is supported by the observation that NP deletion mutant deficient in binding to the 40S ribosome failed to mediate such interaction evident from the lack of strong colocalization between P58^IPK^ and the RPS19 in cells expressing NPΔ151–175 deletion mutant (Fig 5D).

**Fig 6:**
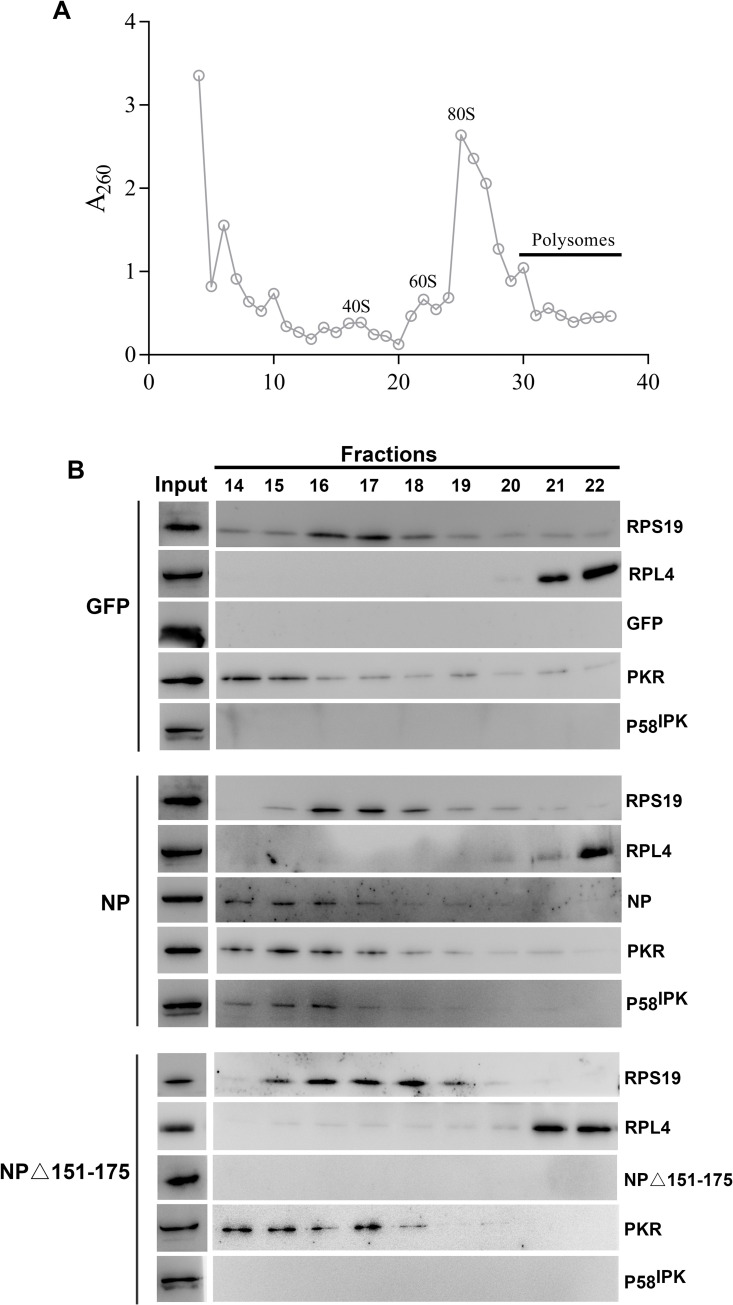
P58^IPK^ cosedimented with 40S ribosomal subunit in the presence of NP. **A**. Ribosomal subunits sucrose density gradient fractionation. Absorbance of sucrose density gradient fractions at 260 nm was plotted verses fraction number. **B**. HEK293T cells were transfected with plasmids expressing EGFP, NP or NPΔ151–175 mutant. The cell lysates were fractionated on sucrose density gradient to generate the ribosomal subunit and polysomes sedimentation diagram, as shown in panel A. Panel B shows the western blot analysis of 40S and 60S ribosomal subunit associated proteins based on the plot diagram in panel A. Selected fractions were mixed with equal volume of 2x SDS sample buffer, boiled and subjected to SDS-PAGE. Input represents the cleared cell lysate. RPS19, RPL4, GFP, PKR, and P58^IPK^ were probed with its specific antibodies. NP and NPΔ 151–175 were probed with anti-Myc tag antibody.

To confirm that NP recruits P58^IPK^ to the 40S ribosomal subunit with the assistance of RPS19, we asked whether P58^IPK^ co-purifies with 40S ribosomal subunit in NP expressing cells. Cell lysates expressing either NP or NPΔ151–175 mutant or GFP, were fractionated on 5–40% sucrose gradient and ribosome sedimentation profiles were recorded based on the absorbance at 260 nm (A260) (Fig 6A). The 40S and 60S ribosomal subunits were monitored in the gradient fractions by the identification of RPS19 and RPL4, the structural components of the 40S and 60S ribosomal subunits, respectively (Fig 6B). The PKR was predominantly found in gradient fractions containing the 40S ribosomal subunit (Fig 6B). Interestingly, the P58^IPK^ co-purified with the 40S ribosomal subunit along with NP and PKR (Fig 6B, middle panel). Such co-purification was not observed from cell lysates expressing either GFP or NPΔ151–175 mutant, which is deficient in binding to the RPS19, a structural component of the 40S ribosomal subunit (Fig 6B, top and bottom panels). This experiment clearly demonstrates that NP with the assistance of RPS19 recruits the P58^IPK^ to the 40S ribosomal subunit where PKR is located.

### P58^IPK^ knockdown induces translational shutoff and restricts Andes virus replication in cells

**Fig 7:**
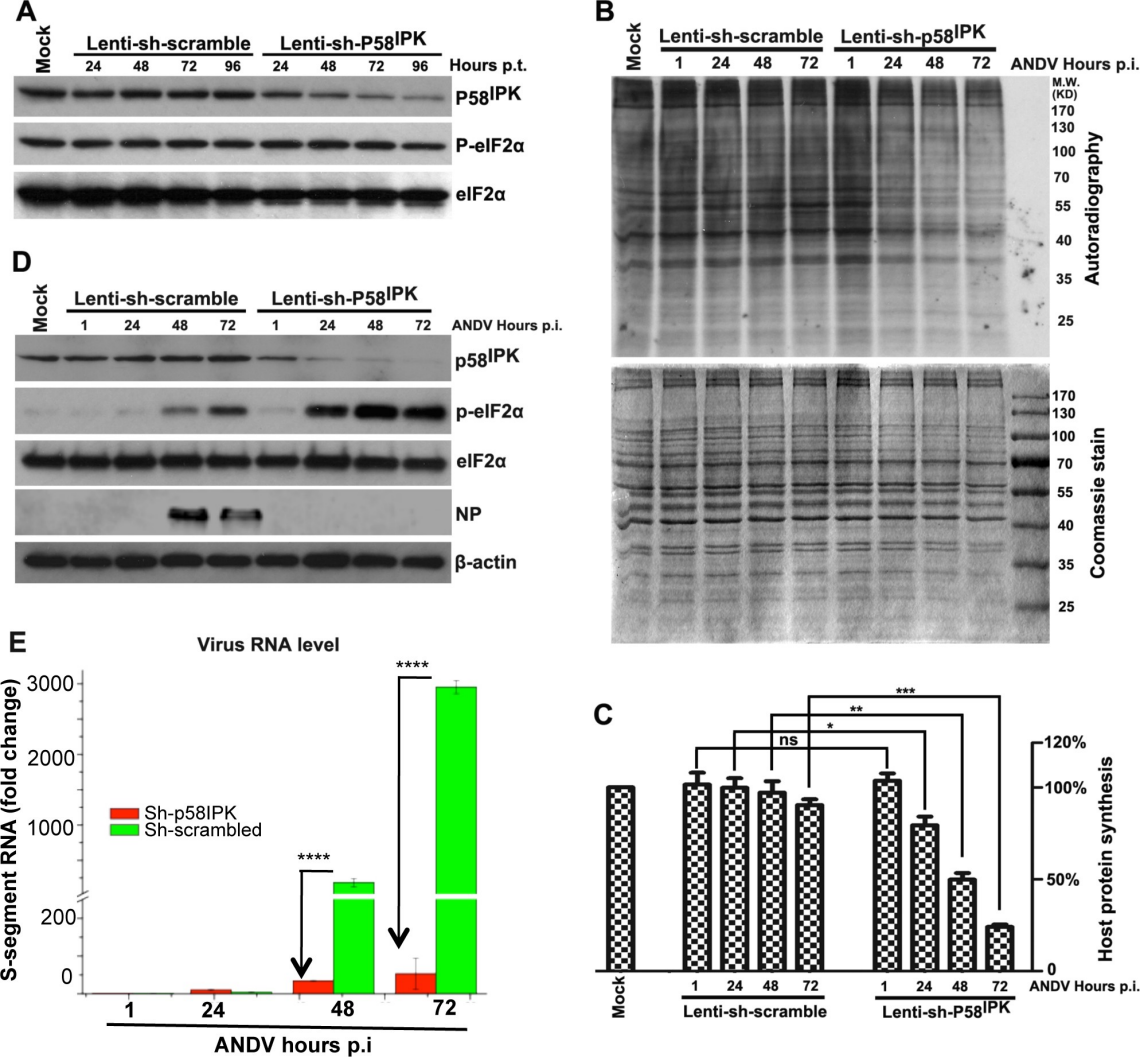
The knockdown of P58^IPK^ induces translational shutoff and restricts Andes virus replication in cells. **A.** HUVECs were transduced with lentivirus at an MOI of 3, expressing either scrambled shRNA or shRNA targeted against P58^IPK^. Cells were harvested at different time points post-transduction and the cell lysates were examined by western blot analysis using appropriate antibodies. **B.** HUVECs were transduced with lentivirus as mentioned in panel A. Cells were infected with Andes virus at an MOI of 0.5, 24 hours post lentivirus transduction. Cells were metabolic labeled with ^35^S methionine-cysteine for 40 min. before harvesting at different time points post Andes virus infection. Cell lysate were separated by SDS-PAGE and visualized by Coomassie blue stain (bottom). The same Coomassie stained gel was dried and exposed to X-ray film (top). **C.** The band intensities from the autoradiogram were quantified by Image-Pro plus software and normalized to that of the mock control. Results from two independent experiments were used to calculate standard deviations, shown as error bars. The significance (*) was calculated by the students *t* test. **D.** The cell lysate from panel B was examined by western blot analysis using appropriate antibodies. **E.** HUVECs were transduced with lentivirus followed by Andes virus infection as mentioned in panel B. Total RNA was purified from cell lysates and viral S-segment RNA levels were quantified in each sample by real time PCR as mentioned in Materials and Methods section. Fold changes in viral RNA levels related to 1.0 hour time point in each group are shown. Results from three independent experiments were used to calculate standard deviations, shown as error bars. The significance in fold change (*) was calculated by the students *t* test.

The inhibition of PKR with the assistance of P58^IPK^ lead to the hypothesis that P58^IPK^ deficient cells would activate PKR upon hantavrius infection and undergo translational shutdown to establish an antiviral state. To test this hypothesis, P58^IPK^ was knockdown in HUVEC cells using lentivirus shRNA delivery system. Since lentivirus transduction at higher MOI induces PKR activation, a careful titration revealed that lentivirus transduction up to an MOI of 3.0 knocks down P58^IPK^ without affecting the phosphorylation of eIF2α (Fig 7A). HUVECs grown in six well plates were transduced with lentivirus, expressing either scrambled or P58^IPK^ specific shRNA. Twenty-four hours post transduction, cells were infected with hantavirus and de-novo protein synthesis was monitored by metabolic labeling at increasing time intervals post infection. It is evident from Fig 7B and 7C that P58^IPK^ deficient cells induced host translation shutoff upon virus infection, which became more effective at increasing time points post infection. An examination of the cell lysates by western blot analysis revealed that P58^IPK^ knockdown triggered the phosphorylation of eIF2α (Fig 7D), causing the shutdown of host translation machinery in virus-infected cells. Further analysis of the cell lysates by real time PCR demonstrated that P58^IPK^ knockdown cells did not efficiently support virus replication. We observed a dramatic decrease in virus replication at 48 and 72 hours post-infection (Fig 7E). Collectively the results from Fig 7 demonstrate that hantaviruses use P58^IPK^ to inhibit PKR and prevent the establishment of antiviral state in virus infected cells.

## Discussion

Activation of PKR-eIF2α pathway by innate immune response during virus infection is primarily aimed to limit viral dissemination in the host by the induction of host translation shutoff and establishment of antiviral state in the host. However, viruses have evolved numerous strategies to counteract this antiviral response. We previously showed that hantavirus NP counteracts PKR antiviral response by inhibiting PKR dimerization using an unknown mechanism. Here we demonstrate that NP activates an endogenous PKR inhibitor P58^IPK^ to inhibit PKR autophosphorylation.

Despite being a PKR inhibitor [38], the P58^IPK^ primarily functions as a molecular chaperone, which is transcriptionally up-regulated during a stress to endoplasmic reticulum (ER) [39,40]. The P58^IPK^ binds to misfolded proteins and aids their proper folding during unfolded protein response (UPR) in the ER [39,40]. In addition, P58^IPK^ inhibits PERK, another eIF2α kinase that regulates protein synthesis during ER stress [39,40]. It has long been thought that P58^IPK^ promotes the translation of influenza virus mRNA by shutting down the PKR antiviral response in virus infected cells by an unknown mechanism [41]. Interestingly, the molecular chaperone Hsp40 forms an inactive complex with P58^IPK^ to restrict its activity [42]. The interaction between P58^IPK^ and PKR is mediated by one of the nine tandemly arranged TPR domains of P58^IPK^ [43]. Also, P58^IPK^ contains a J-domain at its C-terminus, which is required for PKR inhibition *in vivo* [44]. The heat shock protein Hsp70 also forms a complex with P58^IPK^ via its C-terminal J-domain [23]. Similar to other J-domain containing proteins, the P58^IPK^ has been reported to stimulate the ATPase activity of Hsp70 [45]. These observations have led to the hypothesis that P58^IPK^ likely uses Hsp70 to refold PKR and interrupt its dimerization. Interestingly, PKR remains associated with the 40S ribosomal subunit in the cell. While this strategic location likely promotes the easy access of PKR upon activation to its downstream target eIF2α, it remains unclear how P58^IPK^ gains access to this strategic location to interrupt PKR dimerization. Moreover, the lack of molecular mechanism for the release of P58^IPK^ from its negative regulator Hsp40 shows clear gaps in the mechanism of PKR inhibition by P58^IPK^.

Interestingly, NP failed to inhibit PKR autophosphorylation in P58^IPK^ knockdown cells, suggesting that NP requires P58^IPK^ for activity. Further studies revealed that NP binds to P58^IPK^ and the binding site spans to the TPR subdomain I and II at the N-terminus of P58^IPK^. The Hsp40 binding site on P58^IPK^ was mapped to the TRP subdomain II, clearly demonstrating that NP binding site overlaps with the binding site of Hsp40. Since NP binds to P58^IPK^ with higher affinity as compared to Hsp40, it is likely that NP competitively inhibits the binding of Hsp40 to P58^IPK^. The competitive inhibition releases P58^IPK^ from the clutches of Hsp40 and the resulting NP-P58^IPK^ complex was found to interact with the 40S ribosomal subunit via RPS19. Hantaviruses have evolved P58^IPK^ and RPS19 binding domains in NP that enable it to serve as a bridge between P58^IPK^ and the 40S ribosomal subunit. The selective engagement of P58^IPK^ with the 40S ribosomal subunit, with the assistance of NP, facilitates the free access of P58^IPK^ to the PKR. This is supported by the observations that NP mutants lacking either P58^IPK^ or RPS19 binding domains failed to inhibit PKR autophosphorylation in cells. These studies have provided a molecular mechanism by which NP dissociates the P58^IPK^-Hsp40 complex in virus infected cells and recruits the released P58^IPK^ to the 40S ribosomal subunit for the prompt inhibition of PKR. The significance of this unique PKR inhibition strategy in hantavirus replication is evident by the induction of rapid host translation shutoff and establishment of antiviral state in P58^IPK^ deficient cells.

Based on these observations it appears surprising that mammalian cells harbor a PKR inhibitor P58^IPK^ that favors virus replication in the host by the inhibition of PKR antiviral responses. Recent studies have shown that P58^IPK^ also plays a role in the host antiviral defense *in vivo* [38]. PKR activates NF-κB to positively regulate the induction of interferon and inflammatory cytokines [16]. The vigorous inflammatory response due to PKR activation can prove fatal to the host. The rapid PKR activation in P58^IPK^ knockout mice during influenza virus infection undoubtedly controlled the virus replication, however the elevated inflammatory response due to PKR activation caused sever lung pathology and apoptosis that increased the mortality rates in infected mice [38]. These observations suggest that normal P58^IPK^ function would be to calm down PKR antiviral response in the host after virus replication has been controlled. However, hantaviruses exploit P58^IPK^ to prevent PKR activation in virus infected cells to ensure continuous replication without the interruption from PKR antiviral responses.

Due to their small genome size, virus encoded proteins containing multifunctional domains enabling them to interact with numerous host cell factors during the course of viral infection. Hantavirus NP is one of such multifunctional proteins playing diverse roles in hantavirus replication cycle. NP is involved in cap-snatching in conjunction with RdRp [46], translation control of viral mRNAs [47], encapsidation and packaging of viral genome. The RNA helix unwinding activity of NP has been proposed to play a role in genome replication in conjunction with RdRp [48]. The PKR inhibitory function of hantavirus NP further proves the multifunctional nature of viral proteins and the flexibility of their unique domains to interact with numerous host cell factors to support virus replication by combating the multifaceted antiviral defenses of the host cell.

## Materials and methods

### Plasmids

The plasmids pMyc-ANDVNP, pMyc-SNVNP and pMyc-HTNVNP expressing Myc tagged nucleocapsid proteins from Andes virus, Sin nombre virus and Hantan virus were constructed as previously reported [24]. The NP truncation mutants were constructed by PCR amplification and insertion of the corresponding sequence into pcDNA3.1-Myc backbone. The plasmids pMyc-P58^IPK^ and pFLAG-P58^IPK^ were constructed by inserting the P58^IPK^ open reading frame (ORF) into pcDNA3.1-Myc and pcDNA3.1-FLAG backbones, respectively. The P58^IPK^ ORF was amplified from cDNA generated by the reverse transcription of an RNA sample purified from HUVEC cells in culture. The plasmid pGST-P58^IPK^ was cloned by the insertion of P58^IPK^ ORF into pGEX-4T-3 vector. The P58^IPK^ truncation mutants were constructed by PCR amplification and insertion of appropriate gene sequence into pcDNA3.1-Myc backbone. The plasmids pMyc-Hsp40 and pFLAG-Hsp40 were constructed by the insertion of Hsp40 ORF into pcDNA3.1-Myc and pcDNA3.1-FLAG backbones, respectively. The Hsp40 ORF was also amplified from cDNAs generated by the reverse transcription of an RNA sample from HUVEC cells. The plasmid pcDNA3.1 myc his Su(fu) (Addgene plasmid # 13855), expressing the Myc tagged SUFU protein was a gift from Cliff Tabin [49]. The plasmid pet-Hsp40 was constructed by Genscript. The plasmid pLKO.1 - scramble shRNA was a gift from David Sabatini (Addgene plasmid # 1864) [50]. The pLKO.1-P58^IPK^ shRNAs were constructed by inserting the hairpin sequence into pLKO.1 - TRC cloning vector, a gift from David Root (Addgene # 10878) [51]. The target sequences for shRNA knowckdown of P58^IPK^ were: 5’—CAG TCG CAG AAA CGA GAT TAT -3’ and 5’—GAG CCA AGC ATT GCT GAA TAT -3’.

### Cell culture and virus

Human embryonic kidney 293T (HEK293T) and HeLa cells were cultured in Dulbecco’s Modified Eagle’s Medium (DMEM) (Hyclone) supplemented with 10% heat-inactivated fetal bovine serum (Gibco), 2 mM glutamine, 100 U/ml penicillin and 100 μg/ml streptomycin at 37°C under humidified air containing 5% CO2. Human umbilical vein endothelial cells (HUVECs) were purchased from Lonza and cultured in EGM BulletKit medium from Lonza (CC-3124). The propagation of Andes virus (strain Chile-9717869) in cell culture has been previously described [24]. HEK293T stable cell lines were generated by lentivirus infection followed by puromycin selection (3 μg/ml), as previously reported [24]

### Western blot and antibodies

Cells were washed once with phosphate buffered saline (PBS) and lysed with radioimmunoprecipitation assay buffer (RIPA buffer) supplemented with protease and phosphatase inhibitor cocktails (Roche). Clarified cell extracts were mixed with equal volume of 2× SDS loading buffer and boiled at 95°C for 5 min. Proteins were separated by SDS-PAGE and transferred to PVDF membrane (Millipore). The membrane was blocked with 5% non-fat milk in PBST buffer (1x PBS, 0.05% Tween 20) followed by incubating with primary and secondary antibodies diluted in blocking buffer. The blots were developed with ECL (enhanced chemiluminescence) and exposed to X-ray film. The primary antibodies for PKR (12297S), eIF2α(5324S), phosphorylated eIF2α (Ser51)(9721S) and P58^IPK^ (2940S) were from Cell Signaling Technologies. The primary antibody for phosphorylated PKR was from Abcam. The primary antibodies for FLAG tag (F1804), β-actin (A5441) and GFP(G1546) were from Sigma. The primary antibody for c-Myc tag, RPS19(sc-100836) were from Santa Cruz. The primary antibody for Hsp40 (ADI-SPA-400) was from Enzo Life Science. The primary antibody for RPL4(11302-1-AP) was from proteintech. The polyclonal antibody for ANDV and SNV NP was produced in our lab. The antibody for CCHFV NP (ab190657) was from abcam.

### Immunofluorescence staining

HeLa cells grown on coverslips were co-transfected with mCherry-P58^IPK^ along with either Myc-NP or Myc-NP mutants for thirty-six hours using TurboFect Transfection Reagent. Cells were washed once with PBS and fixed with 4% paraformaldehyde in PBS for 15 min at room temperature. The excess paraformaldehyde was neutralized with 0.1 M glycine in PBS, followed by extensive washing with PBS. The fixed cells were permeabilized with 0.5% TritonX-100 in PBS for 5 min and blocked with 3% BSA for 2 hours at room temperature. The cells were sequentially incubated with anti-Myc and Alexa Fluor 488 Goat Anti-Mouse IgG (H+L) antibody (life technologies). The coverslips were mounted in mounting medium containing DAPI (vector labs). The confocal images were recorded with Nikon Digital Eclipse C1 Microscope.

### Coimmunoprecipitation

Coimmunoprecipitation was carried out as previously described [24]. Briefly, HEK293T cells were cotransfected with required plasmids. Cells were lysed with NP-40 lysis buffer (50 mM Tris-HCl pH 7.5, 150 mM NaCl, 0.5% NP-40, 10% glycerol, 1 mM EDTA), supplemented with protease and phosphatase inhibitor cocktails (Roche). Ten percent of the clarified cell lysates were saved as input. The remaining cell lysates were incubated with 1 μg of required antibody for 4 hours at 4°C with gentle rotation. The antigen-antibody complexes were captured with 40 μl of protein G agarose beads (50% slurry) by continuous rotation for one hour at 4°C. The beads were washed four times with lysis buffer, resuspended in 1× SDS loading buffer and boiled at 95°C for 5 min. After brief centrifugation the supernatants were loaded into SDS PAGE gel. Coimmunoprecipitation of HUVEC cell lysates was carried out similarly, except the cells were infected with either Andes virus or hazara virus at an MOI of 1, followed by cell lysis 48 hours post infection.

### Sucrose density gradient fractionation

Ribosomal subunits and polysomes were fractionated on sucrose density gradient. Briefly, HEK293T cells seeded in 10 cm dish were transfected with EGFP, NP or NPΔ151–175 mutant. Cells were treated with Cycloheximide (100 μg/ml final concentration) for 5 min at 48 hours post-transfection. Cells were washed twice with ice-cold PBS containing 100 μg/ml Cycloheximide. Cells were scraped in 5 ml of ice-cold PBS containing 100 μg/ml cycloheximide and centrifuged at 500 xg for 5 min. Cell pellet was resuspended in 500 μl of hypotonic buffer (5 mM Tris-HCl pH 7.5, 2.5 mM MgCl_**2**_, 1.5 mM KCl) supplemented with 1x protease inhibitor cocktail (Roche), 100 μg/ml Cycloheximide, 2 mM DTT, 100 units of RNAase inhibitor, followed by the addition of Triton X-100 and sodium deoxycholate to a final concentration of 0.5% to solubilize ribosomes for 20 min at 4°C. Cell lysates were centrifuged at 16000x g for 15 min at 4°C and supernatants were layered on linear sucrose gradient (5%-40%) centrifuged at 37000 rpm for 3.5 hours at 4°C using SW41 rotor. Fractions of 300 μl sucrose gradient were collected using biocomp fractionator and optical density at 260 nm was measured.

### Protein purification and GST pull-down

GST and GST-P58^IPK^ proteins were expressed in BL21(DE3)pLysS bacterial cells (Novagen). Briefly, the bacteria were grown in 500 ml cultures at 37°C with vigorous shaking until the optical density at 600 nm (OD 600) reached 0.6. The recombinant protein expression was induced by the addition of isopropyl β-D-1-thiogalactopyranoside (IPTG) to the media at a final concentration of 1 mM. The media was shaken at 25°C for additional 5 hours. The bacterial pellets were suspended in lysis buffer (1× PBS, 1mM DTT, 1 mM EDTA, 1% TritonX-100, protease inhibitor cocktail) and sonicated. The clarified cell lysates were loaded on Glutathione HiCap matrix (Qiagen), followed by washing and elution of bound proteins with reduced glutathione following the manufacturer’s instructions. The eluted protein were dialyzed against (1×PBS, 1mM DTT), concentrated and saved at -80°C. For GST pull-down assay, 20 μg of GST or GST-P58^IPK^ were incubated with 50 μl Glutathione HiCap matrix (50% slurry) in 500 μl GST pull-down binding and wash buffer (50 mM Tris·HCl, pH8.0, 200 mM NaCl, 1 mM EDTA, 0.5% Nonidet P-40, 1 mM DTT, supplemented with 1x protease inhibitor cocktail) for 2 hours at 4°C. The Glutathione HiCap matrix was washed and saved as bait. On the other hand, HEK293T cells expressing the proteins of our interest were lysed using GST pull-down binding and wash buffer. The HEK293T cell lysates were clarified and mixed with the bait for one hour at 4°C. The Glutathione HiCap matrix was washed four times with wash buffer and resuspended in 1× SDS loading buffer and boiled at 95°C for 5 min. After brief centrifugation the supernatants were loaded into SDS PAGE gel.

### ^35^S metabolic labeling

Cells grown in 6-well plates were washed twice with starvation medium (DMEM deficient in methionine and cysteine) and cultured in the same medium for 30 min to consume intracellular pools of methionine and cysteine. Cells were then incubated for 40 min with 1 ml of starvation medium containing 300 μCi ^35^S methionine and cysteine (PerkinElmer). Cells were washed with PBS before lysis with 100 μl of RIPA buffer. The cell lysates were mixed with 2× SDS loading buffer, boiled at 95°C for 5 min and separated on 10% SDS-PAGE gel. The gels was stained with coomassie brilliant blue dye, dried on filter paper and exposed to X-ray film over night at -80°C.

### Real time PCR

Total cellular RNA was purified using RNeasy Kit (Qiagen) and reverse transcribed using M-MLV Reverse Transcriptase (invitrogen) according to manufacturer’s instructions. Real time PCR reactions were performed on ABI 7500 real time PCR system (Applied Biosystems), using SYBR green PCR master mix (Roche). Each reaction was performed in triplicates. The mRNA levels of a housekeeping gene β-actin were quantified as an internal control. The relative quantification method was used for data analysis as previously reported [24]. The primers used for the quantification of β-actin mRNA and ANDV S-segment RNA have been reported in our previous publication [24].

### Biolayer interferometry

Biolayer interferometry (BLI) was used to monitor the binding affinities of His tagged NP and HSP40 with GST-P58IPK fusion protein and GST tag using the BLItz system (ForteBio Inc.), as previously reported [28,52,53]. Briefly, the bacterially expressed and purified His tagged NP and HSP40 were loaded onto NiNTA biosensors (Forte Bio Inc.), as previously reported [28,52,53]. All reactions were carried out at room temperature in 1x PBS. After mounting the His tagged protein, the biosensors were equilibrated in 1x PBS and then dipped in the purified protein solutions of either GST-P58IPK or GST at varying concentrations for the measurement of association kinetics. The reaction cycles were as follows: initial base line for 50 seconds, loading of His tagged protein on NiNTA biosensors for 420 seconds, base line for 50 seconds, association of GST-P58^IPK^ and GST with His tagged protein loaded on NiNTA biosensor for 420s, followed by dissociation phase of 600 seconds. The kinetic parameters K_ass_ (association rate constant), K_dis_ (dissociation rate constant) and the binding affinities (Kd = K_dis_/K_ass_) were calculated with the help of inbuilt data analysis software (BLItZ Pro), as previously reported [28,52,53].

### Sucrose density gradient centrifugation

Sucrose gradients were used to examine the oligomeric forms of wild type NP and its deletion mutants. Forty microliters of purified protein containing a C-terminal His tag (110 μM) were layered onto linear gradients containing 5 to 50% (wt/vol) sucrose in 1x PBS and centrifuged at 90,000 rpm in an S120AT3-0098 rotor at 4°C for 6 hours, using a Sorvall Discovery M120SE analytical ultracentrifuge. Gradient fractions of 30 μl were collected. The protein molecular mass markers bovine serum albumin (BSA; 82 kDa), alcohol dehydrogenase (ADH) (150 kDa), and glutathione S-transferase (GST) (27 kDa) were similarly fractionated. Fraction were analyzed by sodium dodecyl sulfate-polyacrylamide gel electrophoresis (SDS-PAGE). The wild type NP and its deletion mutants were analyzed by western blot analysis using anti His-tag primary antibody. The elution profile of GST, BSA and ADH (Fig 4G) was used to determine the oligomerization status of NP and its deletion mutants.
